# Clinical competencies of undergraduate nursing students for labor care: a quasi-experimental study

**DOI:** 10.15649/cuidarte.3679

**Published:** 2024-09-01

**Authors:** Alvar Rafael Castillo-Ramírez, Natanael Librado-González, Hugo Alberto Sánchez-Maldonado, Maritza Nicolas-Santiago, Carolina Urueña-González, Leticia Felipe Herrera

**Affiliations:** 1 Universidad de Chalcatongo, Chalcatongo de Hidalgo, Oaxaca, Mexico. E-mail: kstyo 97@hotmail.com Universidad de Chalcatongo Chalcatongo de Hidalgo Oaxaca Mexico kstyo 97@hotmail.com; 2 Universidad Autónoma de Nuevo León, Facultad de Enfermería, Monterrey, Nuevo León, Mexico, Universidad de Chalcatongo, Chalcatongo de Hidalgo, Oaxaca, Mexico. E-mail: nlibrado3@gmail.com Universidad Autónoma de Nuevo León Universidad Autónoma de Nuevo León Facultad de Enfermería Monterrey Nuevo León Mexico nlibrado3@gmail.com; 3 Universidad de Chalcatongo, Chalcatongo de Hidalgo, Oaxaca, Mexico. E-mail: alberto sm93@hotmail.com Universidad de Chalcatongo Chalcatongo de Hidalgo Oaxaca Mexico alberto sm93@hotmail.com; 4 Universidad de la Sierra Sur, Miahuatlán de Porfirio Díaz, Oaxaca, Mexico. E-mail: maritza.nstgo.unsis@gmail.com Universidad de la Sierra Sur Miahuatlán de Porfirio Díaz Oaxaca Mexico maritza.nstgo.unsis@gmail.com; 5 Universidad de Chalcatongo, Chalcatongo de Hidalgo, Oaxaca, Mexico. E-mail: carouru3@gmail.com Universidad de Chalcatongo Chalcatongo de Hidalgo Oaxaca Mexico carouru3@gmail.com; 6 Universidad de Chalcatongo, Chalcatongo de Hidalgo, Oaxaca, Mexico. E-mail: leticiafelipeherrera@gmail.com Universidad de Chalcatongo Chalcatongo de Hidalgo Oaxaca Mexico leticiafelipeherrera@gmail.com

**Keywords:** Quasi-experimental Study, High Fidelity Simulation Training, Students, Nursing, Competency-Based Education, Labor, Obstetric, Estudios Cuasi Experimentales, Enseñanza Mediante Simulación de Alta Fidelidad, Estudiantes de Enfermería, Educación Basada en Competencias, Trabajo de Parto, Estudos Quase-Experimentais, Treinamento com Simulação de Alta Fidelidade, Estudantes de Enfermagem, Educação Baseada em Competências, Trabalho de Parto

## Abstract

**Introduction:**

Clinical simulation in the management of labor improves undergraduate nursing students' clinical competencies by integrating knowledge, skills, and attitudes. This integration facilitates the internalization of theoretical knowledge, reinforcing self-esteem and confidence in providing care.

**Objective:**

To evaluate the effect of clinical simulation on the development of clinical competencies for the management of labor in undergraduate nursing students at a public university in Oaxaca.

**Materials and Methods:**

A quasi-experimental pre- and post-test study was conducted with 38 undergraduate nursing students, men and women, in the sixth (18) and fourth (20) semesters. The sample size for comparison of means was calculated in G*Power 3. Data were collected using the Clinical Simulation Competency Assessment Tool (ClinSimCAT). Descriptive and inferential statistical analysis was performed using the statistical software SPSS version 26.0.

**Results:**

The Wilcoxon test revealed statistically significant differences between the mean pretest and post-test scores for the Intervention Group (IG) (p < 0.001). In addition, the Student's t-test showed significant differences between the means of the IG and Control Group (CG) in the post-test (t = 7.598, df = 37, p < 0.0001).

**Discussion:**

Clinical simulation significantly improved students' clinical competencies in the management of labor, which is consistent with the findings of other research studies.

**Conclusion:**

It is crucial that clinical simulation is not limited to technical procedures but also promotes the development of comprehensive nursing skills.

## Resumen.

**Introducción:**

La simulación clínica en el manejo de trabajo de parto mejora las competencias clínicas de los estudiantes de pregrado en enfermería al integrar conocimientos, habilidades y actitudes. Esta integración facilita la interiorización de los conocimientos teóricos, reforzando la autoestima y la confianza en los estudiantes.

**Objetivo:**

Evaluar el efecto de la simulación clínica en el desarrollo de competencias clínicas para el manejo del trabajo de parto en estudiantes de pregrado en enfermería de una universidad pública de Oaxaca.

**Materiales y Métodos:**

Estudio cuasiexperimental pretest y postest con 38 estudiantes de pregrado en enfermería, hombres y mujeres, de sexto (18) y cuarto (20) semestre, la muestra para comparación de medias se calculó en G*Power 3. Los datos se recolectaron con el instrumento "Evaluación de Competencias en Simulación Clínica" (ClinSimCAT). Se realizó análisis estadístico descriptivo e inferencial a través del software estadístico SPSS versión 26.0.

**Resultados:**

La prueba de Wilcoxon reveló diferencias estadísticamente significativas entre las puntuaciones medias del pretest y postest para el Grupo Intervención (GI) (p < 0,001). Además, la prueba t de Student mostró diferencias significativas entre las medias del GI y Grupo Control (GC) en el postest (t = 7.598, gl = 37, p < 0.0001).

**Discusión:**

La simulación clínica mejoró significativamente las competencias clínicas de los estudiantes en el manejo del trabajo de parto, esto concuerda con los hallazgos en otras investigaciones.

**Conclusión:**

Es crucial que la simulación clínica no se limite a procedimientos técnicos, sino que también promueva el desarrollo de habilidades integrales en enfermería.

## Resumo.

**Introdução:**

A simulação clínica na gestão do trabalho melhora as competências clínicas dos estudantes de graduação em enfermagem ao integrar conhecimentos, habilidades e atitudes. Essa integração facilita a internalização do conhecimento teórico, reforçando a autoestima e a confiança nos alunos.

**Objetivo:**

Avaliar o efeito da simulação clínica no desenvolvimento de competências clínicas para a gestão do parto em estudantes de graduação em enfermagem de uma universidade pública de Oaxaca.

**Materiais e Métodos:**

Estudo quase-experimental pré-teste e pós-teste com 38 estudantes de graduação em enfermagem, homens e mulheres, do sexto (18) e quarto (20) semestre, a amostra para comparação de médias foi calculada no G*Power 3. Os dados foram coletados com o instrumento “Avaliação de Competências em Simulação Clínica” (ClinSimCAT). A análise estatística descritiva e inferencial foi realizada por meio do software estatístico SPSS versão 26.0.

**Resultados:**

O teste de Wilcoxon revelou diferenças estatisticamente significativas entre as médias dos escores do pré e pós-teste para o Grupo Intervenção (GI) (p < 0,001). Além disso, o teste t de Student mostrou diferenças significativas entre as médias do GI e do Grupo Controle (GC) no pós-teste (t = 7,598, gl = 37, p < 0,0001).

**Discussão:**

A simulação clínica melhorou significativamente as competências clínicas dos estudantes na gestão do trabalho de parto, o que está de acordo com os resultados de outras investigações.

**Conclusão:**

É fundamental que a simulação clínica não se limite a procedimentos técnicos, mas também promova o desenvolvimento de competências abrangentes de enfermagem.

## Introduction

Clinical competencies are defined as "the ability of a nurse to demonstrate mastery of the knowledge, skills, and attitudes"^1^ necessary to make informed decisions and implement effective interventions in clinical situations. They are essential to guaranteeing quality patient-centered care. These competencies include teamwork and collaboration, quality of care, use of informatics and technology, communication, systems-based practice, and professionalism to ensure quality maternal-child care^2^^, ^^3^.

Currently, Maternal-Child Nursing education faces several challenges in training future professionals in the clinical setting^4^, such as unsystematic curriculum monitoring, inadequate resources, improvement of the learning environment and clinical supervision, lack of student motivation, and the need to improve communication skills and update teaching methods^5^. In this sense, it is important to implement active teaching methods that allow nursing students to play a crucial role in the care of women during pregnancy, childbirth, and the puerperium, as well as in the newborn’s health^5^, coordinating care among team members, communicating the results of the assessment in the delivery process, providing physical and emotional support, as well as pain management and monitoring the health of both the mother and the newborn^6^.

Undoubtedly, clinical simulation (CS) practice and the use of technology are among the most effective contemporary methods for nursing students to develop clinical competencies. This integration is essential for strengthening nursing as a profession that combines art and science in the care of women in labor, providing a comprehensive education that integrates theoretical knowledge with practical skills and attitudes in the clinical setting^2^^, ^^4^. As a result, CS is a highly active and effective teaching method. It is defined as a controlled representation of reality that mimics a real environment through scenarios and techniques that involve fully guided interactive experiences.^7^ In addition, CS has acquired a prominent role in the education of nursing students due to its potential benefits in terms of patient safety, ethics of care, improvement of technical- cognitive knowledge, development of self-confidence, and clinical competence in various contexts. This active teaching method ensures that students are better able to deal with real-life situations and provide effective and competent care to women in labor^8^^, ^^9^^, ^^10^.

In addition, several studies highlight the importance of CS tools in improving outcomes and reducing complications associated with induction and conduction of labor. Schneider emphasizes the importance of realistic simulations to train obstetricians in forceps-free extractions, which help avoid unnecessary cesarean deliveries^11^. Ami et al. ^12^ demonstrate that incorporating birth simulation software into decision-making processes can significantly reduce emergency cesarean sections and instrumental deliveries, thereby improving the allocation between planned cesarean sections and trial of labor. Morchi et al. ^13^ introduced a new childbirth platform with real-time monitoring of fetal head position, which aids in proper labor management and improves skills through simulation-based training. Additionally, Hashem et al. ^14^ and Yu^15^ highlight the effectiveness of high-fidelity simulation training programs in improving students' clinical competence, knowledge, and confidence in managing the third stage of labor, ultimately preventing complications such as postpartum hemorrhage and improving clinical practice skills.

A scoping and systematic review to identify and map valid and reliable tools used to assess safety in nursing simulation experiences highlighted several tools used to assess technical skills and safety. Most of these tools consist of holistic rubrics or binary skill checklists^16^^, ^^17^. Among these tools is the Clinical Simulation Competency Assessment Tool (ClinSimCAT), which was designed as a comprehensive assessment tool that includes safety as a key competence^18^^, ^^19^. However, to effectively implement the ClinSimCAT, it is necessary to assess students at three levels, following the novice-to-expert model proposed by Benner^20^. This model, which ranges from novice to expert level, provides a framework for developing nursing competencies. Competencies assessed include patient-centered care, teamwork and collaboration, evidence-based practice, quality improvement, safety, professionalism, and systems-based practice^21^^, ^^24^.

In the first three Benner's stages of proficiency^20^ (on which the study is based), the criteria of novice, advanced beginner, and competent are used to measure the students’ performance level using the ClinSimCAT. Table 1 describes the levels of clinical competence.

**Table 1:** Clinical competence levels based on Benner.

Level	Description
Level 1 (Novice)	Students lack the confidence to provide safe care to women in labor and require verbal and physical instruction. If learners are at this level, it indicates that they have not met the clinical/ simulation objectives in the care of women in labor.
Level 2 (Advanced beginner)	Students show satisfactory performance and require intermittent assistance with instructions for the care of women in labor. Their behaviors are independent and/or in response to occasional external guidance. When students are at this level, it indicates that they have met the clinical/simulation objectives in the care of women in labor.
Level 3 (Competent)	Students are more confident, efficient, and coordinated in their actions when caring for women in labor. Their behaviors are independent, competent, and consistent. At this level, the students have met the clinical/simulation objectives in the care of women in labor.

*Source: Adapted from Benner*^18^^, ^^20^*.*

It is worth noting that students with greater self-confidence are more likely to succeed in their interventions because they can test and apply their skills more easily, face new challenges, and overcome failure more quickly. In addition, student satisfaction with simulated experiences is a crucial point to evaluate and consider, as it positively correlates with increased motivation in the teaching learning process^25^^, ^^26^.

In light of the above, this study's rationale lies in the importance of incorporating technology for educational advancement and the adoption of new pedagogical approaches in the teaching learning process. It also emphasizes the need for substantial evidence in the literature to support the investment in robotic equipment for the practice of CS in institutions dedicated to training new nursing human capital.

Given this scenario, the objective of this study is to evaluate the effect of CS on the development of clinical competencies for the management of labor by undergraduate nursing students at a public university in Oaxaca. The hypothesis states that there will be a significant difference in the level of clinical competence in labor care between students who participate in the Clinical Simulation Methodology (CSM) and those who do not.

## Materials and Methods

### A Study design

A quasi-experimental design study was conducted with a non-equivalent control group (pretest and posttest). It included two groups of participants^27^ from a rural public university in Oaxaca, Mexico, during March 2022. This design allowed a comparison of the CS intervention between the IG and the CG. G*Power software was used to calculate the sample size for the comparison of means with a 0.05 error level, a 95% confidence level, and an effect size of 0.5. Furthermore, the accessibility and availability of the participants in the already formed groups of the educational institution were considered.

### Participants

Participants were selected based on the following inclusion criteria: male and female students enrolled in the institution in the sixth and fourth semesters, not failing or repeating any subjects in the semester, having taken and passed Maternal Child Nursing and Gynecological Obstetrics. Students from other undergraduate programs, first- and second-year students, and students with previous experience in labor and delivery were excluded. Students who did not meet 100% of the minimum requirements to participate in the study were excluded.

**Figure 1:**
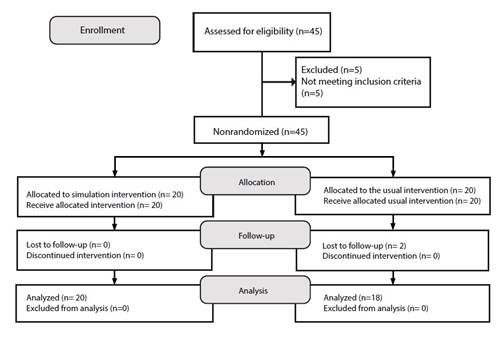
Transparent Reporting of Evaluations with Nonrandomized Designs (TREND) Flowchart.

The IG and CG were formed as follows: for the IG, 20 fourth-semester students who received the SC intervention were selected, and for the CG, 20 sixth-semester students who did not receive the intervention were selected. Figure 1 shows the Transparent Reporting of Evaluations with Nonrandomized Designs (TREND) flowchart^28^.

### Study setting

The study was conducted at the Robotic Clinic of a rural public university and addressed three clinical scenarios of labor care: Admission to the emergency department with transfer to the labor ward, labor in the labor ward, and delivery room. These scenarios were meticulously designed by experts according to the institutional criteria of the International Nursing Association for Clinical Simulation and Learning (INACSL) ^29^ and reviewed by three maternal-child experts using a checklist^30^.

It is important to emphasize that the Robotic Clinic of the educational institution provided all the materials, biomedical equipment, and high and medium-fidelity simulators. The scenarios featured realistic environments, materials, and equipment to simulate labor care^31^. To represent a pregnant woman, the Gaumard® NOELLE® S551.250 high-fidelity simulator programmed with physiological parameters for eutocia according to the clinical case and nursing history designed for the intervention was used.

### Intervention and procedure

The intervention was designed according to CSM and INACSL standards^29^ and lasted 4 hours and 55 minutes. It was divided into 145 minutes of lectures on labor and 150 minutes of lectures on the care of women in labor using the CSM exclusively for the IG.

Activities were conducted in four phases: 1) theoretical training, 2) pretest evaluation, 3) intervention with the clinical simulation program, and 4) posttest evaluation.

In the first phase, both the IG and the CG received theoretical training on work care under the guidance of a traditional teaching program conducted by the subject’s head professor. In the second stage, only the IG underwent a pretest evaluation using a CS scenario evaluated with ClinSimCAT^18^ to determine the level of competencies acquired only with the traditional teaching program. In the third phase, the IG participated in a CS teaching program, which included a detailed guide with the following sections: name of the course, name of the clinical scenario, authors, participants, location and date, schedule, objectives, scenario description, involved personnel, key points, scenario preparation, scenario development and data for the simulator, participants’ attitude, and debriefing.

**Figure 2:**
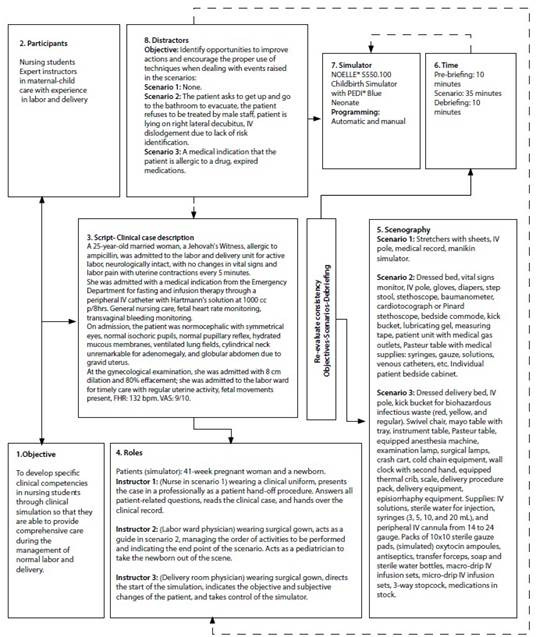
CS Scenario Template for management of labor.

Finally, in phase four, a posttest evaluation of the IG and CG was conducted using the ClinSimCAT and the simulation scenario to determine the IG’s development and level of competencies after implementing the CS teaching program. It should be noted that the CG had previously been exposed to the teaching methodology used by the university, which included theoretical classes, clinical practice in hospitals, and traditional procedural practices. For this reason, it was decided that a pretest evaluation should not be conducted in the CG. Figure 2 details the eight steps for developing the CS scenario for management of labor: 1. Objective, 2. Participants, 3. Script (clinical case description), 4. Roles, 5. Setting, 6. Timing, 7. Simulators, and 8. Distractors. ^31^

### Measurement instrument

The instrument was divided into two sections. The first section is the sociodemographic data questionnaire, which includes variables such as sex, age, semester, religion, and indigenous language spoken. The second section corresponds to ClinSimCAT^18^, a tool designed to be used in clinical or simulation settings in various nursing courses, including labor and delivery in the maternal-child field (https://drive.google.com/file/d/12nDD6wSf0KVKwoyQkTxjXfNY4yybvR9/view?usp=sharing). This instrument assesses the level of competence of students and consists of 20 competencies divided into eight specific domains: 1) patient-centered care, 2) teamwork and collaboration, 3) evidence-based practice, 4) quality improvement, 5) safety, 6) informatics, 7) professionalism, and 8) system-based practice^18^. Below is a brief description of each domain, with the competence number in parentheses.

- *Domain 1:* Patient-centered care (6) involves interactions that focus on the patient's physical, mental, emotional, and spiritual well-being while maintaining professional boundaries.
- *Domain 2:* Teamwork and collaboration (4) refers to interdisciplinary care in which nurses, physicians, social workers, and therapists share information and work together to care for the patient.
- *Domain 3:* Evidence-based practice (1) involves incorporating clinical guidelines and relevant studies into nursing practice.
- *Domain 4:* Quality improvement (1) focuses on finding ways to provide better patient care and services, as well as improving the work environment.
- *Domain 5:* Safety (1) involves maintaining precautions and protocols to identify and manage potential problems.
- *Domain 6:* Informatics (1) includes using electronic medical records and other technologies for medication administration, diagnosis, and care planning.
- *Domain 7:* Professionalism (5), as evidenced by communication, hygiene, attire, interactions, preparation for clinical simulation, justification of actions, and demonstration of knowledge.
- *Domain 8:* Systems-based practice (1) refers to the use and management of resources, supplies, medications, equipment, and information to provide care effectively.

A minimum of 20 and a maximum of 60 points were considered. In domains 3, 4, 5, 6, and 8, each consisting of a single competence, the following scores were assigned: 1 point for Level 1, 2 points for Level 2, and 3 points for Level 3. For domains 1, 2, and 7, which cover 6, 4, and 5 competencies, respectively, the ranking was as follows: Domain 1: 6-9 points for Level 1, 10-13 points for Level 2, and 14-18 points for Level 3; Domain 2: 4-6 points for level 1, 7-9 points for Level 2, and 10-12 points for Level 3; and Domain 7: 5-8 points for Level 1, 9-12 points for Level 2, and 13-16 points for Level 3. As for the general classification of the student's performance level, the following scores were established: Level 1 (novice): 20-32 points, Level 2 (advanced beginner): 33-45 points, and Level 3 (competent): 46-60 points.

The ClinSimCAT was content-validated by experts in nursing, university teaching, and neuropsychology to ensure its relevance and validity within the cultural and linguistic context, whit a Cronbach's alpha of 0.91.

### Ethical considerations

This study adheres to the ethical regulations set forth in the General Health Law's regulations on health research^32^. The participants’ dignity, human rights and welfare are respected in accordance with the principles of autonomy, non-maleficence, and justice. The study was approved and authorized by the educational institution (UNICHA/0186/2020). Participants were gathered in a classroom and informed consent was given, explaining the process of participation and withdrawal in the event of health risk or if they wished to discontinue participation.

### Data analysis

Raw data were stored in Mendeley Data^33^ and analyzed descriptively and inferentially using the Statistical Package for the Social Sciences (SPSS) version 26.0^34^. Descriptive statistics were used to examine sociodemographic data and pretest and posttest scores. Two types of analyses were performed to evaluate the differences between IG and CG: a nonparametric analysis using the Wilcoxon signed-rank test and a parametric analysis using the Student's t-test. The choice of statistical hypothesis tests was based on the assessment of the normality of the data using the Shapiro-Wilk test.

## Results

The study initially included 45 participants, 21 fourth-semester and 24 sixth-semester Bachelor of Science in Nursing students enrolled in the 2021-2022 B semester. Of the 45 eligible nursing students, five were excluded according to the established criteria, 40 participated in the study, and two of them dropped out of CG. The range of age variation in IG was 19 to 31 years with a mean age of 20.90 (SD=2.78), and in CG was 20 to 28 years with a mean of 21.56 (SD=1.85). The sociodemographic characteristics of the participants are detailed in Table 2.

**Table 2:** GI and GC sociodemographic data.

Participants Variable	IG 52.63 (20) %*(f)*	CG 47.37 (18) %*(f)*
Sex		
Female	80.00 (16)	75.00 (15)
Male	20.00 (4)	15.00 (3)
Years (MD ± SD)	20.90 ± 2.78	21.56 ± 1.85
Religion		
Catholic	85.00 (17)	100.00 (18)
None	15.00 (3)	0
Speaker of an indigenous language		
Mixtec	25.00 (5)	16,66 (3)
Triqui	0	5,55 (1)
None	75.00 (15)	77,77 (14)
Evaluations		
Pretest (M/MD)	30.10 ± 4.78	
Posttest (M/MD)	41.50 ± 5.44	30.50 ± 3.12

*Note: f = frequency, % = percentage, M = mean, MD = median, SD = standard deviation, IG = intervention group, CG = control group.*

Table 3 presents the results by competence domains. In the IG, the pretest evaluation shows that few students achieved Level 3, excelling only in the informatics domain. Most students scored at lower levels in other domains. Following the implementation of the CSM, a significant performance improvement was observed, with more than 55% of students scoring at Level 2 in all assessed domains and an increase in the percentage of students reaching Level 3 in several domains. Comparing the posttest results between the IG and the CG, it was found that the IG had a higher percentage of students at Level 3 in five domains, whereas the CG excelled only in systems-based practice and had a significant percentage of students at lower levels in several domains. More than 50% of IG students scored at Level 2 in all domains, while CG students showed similar percentages in only two domains. Finally, the IG had fewer students at Level 1 than the CG, where six domains had percentages above 40% of students at this level.

**Table 3:** IG pretest and posttest performance and CG posttest performance by competence domain.

Domain and competence level	**GI-Pretest (20) %*(f)***	**GI-Posttest (20) %*(f)***	**GC- Posttest (18) %*(f)***
Domain 1: Patient-centered care			
Novice	75.00(15)	10.00(2)	83.33 (15)
Advanced Beginner	25.00(5)	55.00(11)	16.66 (3)
Competent	0	35.00(7)	0
Domain 2: Teamwork and collaboration			
Novice	25.00 (5)	5.00 (1)	55.60 (10)
Advanced Beginner	75.00 (15)	65.00 (13)	44.40 (8)
Competent	0.00 (0)	30.00 (6)	0.00 (0)
Domain 3: Evidence-based practice			
Novice	65.00 (13)	10.00 (2)	50.00 (9)
Advanced Beginner	35.00 (7)	90.00 (18)	50.00 (9)
Competent	0 (0.00)	0.00 (0)	0.00 (0)
Domain 4: Quality improvement			
Novice	70.00 (14)	25.00 (5)	44.40 (8)
Advanced Beginner	30.00 (6)	75.00 (15)	55.60 (10)
Competent	0.00 (0)	0.00 (0)	0.00 (0)
Domain 5: Safety			
Novice	30.00 (6)	5.00 (1)	61.10 (11)
Advanced Beginner	70.00 (14)	85.00 (17)	38.90 (7)
Competent	0.00 (0)	10.00 (2)	0.00 (0)
Domain 6: Informatics			
Novice	35.00 (7)	5.00 (1)	50.00 (9)
Advanced Beginner	55.00 (11)	80.00 (16)	44.40 (8)
Competent	10.00 (2)	15.00 (3)	5.60 (1)
Domain 7: Professionalism			
Novice	85.00 (17)	10.00 (2)	100.00 (18)
Advanced Beginner	15.00 (3)	90.00 (18)	0.00 (0)
Competent	0.00 (0)	0.00 (0)	0.00 (0)
Domain 8: Systems-based practice			
Novice	40.00 (8)	5.00 (1)	0.00 (0)
Advanced Beginner	60.00 (12)	85.00 (17)	5.60 (1)
Competent	0.00 (0)	10.00 (2)	94.40 (17)

*Note: nIG (20), nCG (18), f = frequency, % = percentage.*

When the Wilcoxon signed-rank test was performed to compare IG pretest and posttest medians, it revealed a significant difference (p < 0.001).

**Figure 3:**
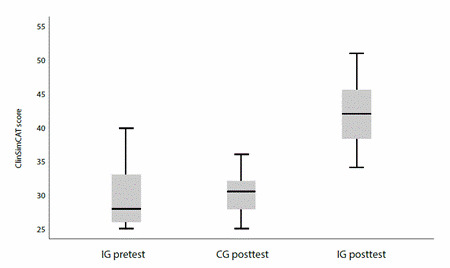
Distribution of GI pretest-posttest scores and GC posttest scores

Finally, the Student's t-test for independent samples was used to compare the means of IG and control group (CG) in the posttest measurement. The results showed statistically significant differences (t = 7.598, gl = 37, p < 0.001). This suggests that students who participated in the CSM program significantly improved their clinical competencies in labor and delivery care compared to those who did not participate (Figure 3).

## Discussion

The results of this study highlight the effectiveness of CS in developing nursing students' competencies, especially in the management of labor^4^^, ^^10^. The comparison of IG pretest and posttest results revealed significant improvements in all areas assessed, confirming the initial hypothesis of the study. These findings are consistent with those reported by Pajohideh et al. ^35^ regarding CS-based training as preparation prior to formal clinical education, demonstrating a sustained effect and improvement in students' skills in caring for women in normal vaginal delivery.

It was noted that CS provides a safe and controlled environment where students can practice and hone their skills without putting actual patients at risk. This aspect is fundamental to developing clinical skills, as it allows students to face complex and high-pressure situations realistically, improving their ability to respond and make decisions in the clinical setting^2^. In this regard, nursing students face several challenges in the clinical learning environment, such as anxiety, lack of confidence, and time constraints, which are significant barriers to effective learning.

Rusch et al.^1^ and Akyüz^5^ report that CS implementation can mitigate these challenges by providing a space where learners can repeatedly practice their skills and receive immediate feedback without the stress of adversely affecting an actual patient. Additionally, it allows students to face a variety of clinical scenarios, from common situations to complex emergencies, in an environment where mistakes are learning opportunities rather than adverse events. The comparison between IG and CG also showed statistically significant differences. This suggests that students who participated in the CSM not only improved their clinical competencies, but also outperformed those who did not participate in the intervention. This finding is consistent with previous studies that have demonstrated the benefits of CS in health education, improving students' self-confidence, technical knowledge, and communication skills when providing care to women in labor^14^^, ^^36^.

It is important to note that competence improvement was not uniform across all domains. The greatest progress was made in the 'teamwork and collaboration,' 'safety,' and 'patient-centered care' domains. These results underscore the importance of CS not only for developing technical skills, but also for fostering teamwork and effective communication, essential elements for safe, high-quality clinical practice^29^. The ‘professionalism’ domain also showed significant improvements, reflecting the impact of CS on the formation of professional attitudes and behaviors. This is critical in maternal-child care, where empathy, respect, and ethics are central to the nurse-patient relationship^2^.

Despite these positive results, the study has some limitations that should be considered. The use of a quasi-experimental design and the sample size limited the generalizability of the results. In addition, the evaluation focused on a single academic and geographic setting that may not reflect the diversity of other educational contexts. Although the ClinSimCAT demonstrated good content validity in the study, further research on its internal consistency as well as concurrent and predictive validity testing with larger and more diverse samples would be beneficial. It would also be valuable to determine whether competencies are culturally influenced and thus assess the feasibility of ClinSimCAT in different contexts.

Future research should consider randomized experimental designs and expand the sample to include diverse institutions and contexts. It would also be beneficial to explore the long-term effects of CS in actual clinical practice, as well as its impact on other nursing and healthcare fields.

## Conclusion

This study provides robust evidence of the positive impact of CS on the clinical competence education of nursing students, particularly in labor and delivery care. CS emerges as a valuable methodology for cultivating students' competencies, proving particularly useful in enhancing the competencies of those with no previous experience with actual patients and suggesting a promising outlook for their future workplace performance. Likewise, CS allows for the improvement of skills and attitudes through repetition and feedback without risk to patients, reinforcing its positive impact on student's academic training. Therefore, implementing SC programs is an effective strategy for improving nursing education, ensuring that future professionals are better prepared to meet the challenges of clinical practice and provide high-quality, patient-centered care.

Finally, to optimize this practice, we recommend integrating a theoretical class on the subject, considering prior knowledge, implementing pre-briefing and briefing sessions, creating clinical simulation scenarios with clinical cases, and conducting debriefing. In addition, it is essential to contextualize CSM in various topics of the nursing curriculum, presenting it as a practical method of teaching and not as a substitute for practice in real settings.
